# Adenosine/TGFβ axis in regulation of mammary fibroblast functions

**DOI:** 10.1371/journal.pone.0252424

**Published:** 2021-06-08

**Authors:** Georgii Vasiukov, Anna Menshikh, Philip Owens, Tatiana Novitskaya, Paula Hurley, Timothy Blackwell, Igor Feoktistov, Sergey V. Novitskiy

**Affiliations:** 1 Department of Medicine, Division of Allergy, Pulmonary, Critical Care Medicine, Vanderbilt University Medical Center, Nashville, TN, United States of America; 2 Department of Medicine, Division of Nephrology, Vanderbilt University Medical Center, Nashville, TN, United States of America; 3 Department of Pathology, University of Colorado Boulder, Denver, CO, United States of America; 4 Department of Pathology, Microbiology and Immunology, Vanderbilt University Medical Center, Nashville, TN, United States of America; 5 Department of Medicine, Division of Hematology/Oncology, Vanderbilt University Medical Center, Nashville, TN, United States of America; 6 Department of Medicine, Division of Cardiovascular Medicine, Vanderbilt University Medical Center, Nashville, TN, United States of America; Peter MacCallum Cancer Centre, AUSTRALIA

## Abstract

Cancer associated fibroblasts (CAF) play a key role in cancer progression and metastasis. Diminished TGFβ response on CAF correlates with poor outcome and recurrence in cancer patients. Mechanisms behind lost TGFβ signaling on CAF are poorly understood, but, utilizing MMTV-PyMT mouse model, we have previously demonstrated that in tumor microenvironment myeloid cells, producing adenosine, contribute to downregulated TGFβ signaling on CAFs. In the current work, we performed serial *in vitro* studies to investigate the role of adenosine/TGFβ axis in mouse mammary fibroblast functions, i.e., proliferation, protein expression, migration, and contractility. We found that adenosine analog NECA diminished TGFβ-induced *CCL5* and *MMP9* expression. Additionally, we discovered that NECA completely inhibited effect of TGFβ to upregulate αSMA, key protein of cytoskeletal rearrangements, necessary for migration and contractility of fibroblasts. Our results show that TGFβ increases contractility of mouse mammary fibroblasts and human fibroblast cell lines, and NECA attenuates theses effects. Using pharmacological approach and genetically modified animals, we determined that NECA effects on TGFβ pathway occur *via* A_2A_/A_2B_ adenosine receptor—AC—PKA dependent manner. Using isolated CD11b^+^ cells from tumor tissue of CD73-KO and CD39-KO animals in co-culture experiments with ATP and AMP, we confirmed that myeloid cells can affect functions of mammary fibroblasts through adenosine signaling. Our data suggest a novel mechanism of interaction between adenosine and TGFβ signaling pathways that can impact phenotype of fibroblasts in a tumor microenvironment.

## Introduction

It is recognized that the tumor microenvironment (TME), comprising of different cell types and extracellular matrix (ECM), can determine the tumor fate [1, 2]. It has been shown that ECM plays a crucial role in the tumor progression, mediates invasion, intra- and extravasation of the tumor cells, and can impact anti-tumor immune response and the efficacy of anti-tumor therapy [3]. ECM in the tumor microenvironment can be assessed by different modalities: bulk and cell-specific gene arrays can demonstrate early changes in ECM-related genes (*Col I*, *αSMA*, *LOX*, etc.), providing mechanistical insights [4]; while measurement of accumulated proteins (collagen, fibronectin, laminin and etc.) can delineate temporal tumor-host interactions [5]; physical properties of ECM, such as elasticity and stiffness [6–8], allows for measurement of ECM mechanical function that shown to contribute to tumor aggressiveness [9–12]. Fibroblasts play a key role in the formation and remodeling of ECM structures in TME [13–15]. In addition to this well-defined function, in recent years it was recognized that fibroblasts contribution to TME is more complex and dynamic with evidences for both tumor-promoting and tumor-suppressive actions [16]. These actions are mediated both by cell-cell contacts as well as by secreted factors [3].

Transforming growth factor-beta (TGFβ) family members, including TGFβ, activin/inhibin and bone morphogenic proteins (BMPs) participate in a wide variety of biological processes under normal and pathological conditions [1]. Proteins of the TGFβ family are primarily morphogens and play a crucial role in the embryonic development and adult tissue regeneration and homeostasis [17]. Dysregulation, such as mutations in the TGFβ signaling pathway, is frequently associates with cancer [18, 19]. TGFβ broad biological effects include regulation of cell proliferation, differentiation, motility, apoptosis, immune cell functions, ECM remodeling, and tumor cell invasion and metastasis. The action of TGFβ is context dependent, which makes the interpretation of its biological effects difficult and requires consideration of microenvironmental factors such as cell type, developmental stage and interaction with other signaling cascades [20]. Indeed, at the early stages of carcinogenesis, TGFβ signaling in epithelial cells is tumor suppressive, at later stages, however, it promotes the invasion and metastasis of neoplastic cells [21]. In myeloid cells, a lack of TGFβ signaling decreases tumor size and the number of metastases [22–24], whereas in fibroblasts, decreased TGFβ signaling correlates with poor prognosis in cancer patients [25]. While TGFβ demonstrates a strong impact on the main functions of fibroblasts, including proliferation, cytokine secretion, cytoskeletal structure rearrangement and ECM deposition and remodeling [20, 26–28], its actions on fibroblasts within complex and dynamic tumor microenvironment, where it can crosstalk with other signaling pathways, still not fully dissected.

Previously, our group has shown that TGFβ signaling on myeloid cells regulates expression and activity of CD73 (Ecto-5′-nucleotidase) and affects tumor progression [22]. CD73 is a membrane protein that, downstream of CD39 (ectonucleoside triphosphate diphosphohydrolase-1), forms a molecular tandem hydrolyzing extracellular ATP and ADP into adenosine. ATP/ADP and adenosine are potent modulators of cell functions, acting in auto- and paracrine manner [29]. Adenosine has been implicated in many pathological processes [30] and is one of the hallmarks of tissue hypoxia [31], including in cancer [32, 33]. Adenosine exhibits pro-tumorigenic effects [34] in the microenvironment through four types of membrane receptors: A1, A2a, A2b and A3 [35, 36]. In our recent work we have suggested a novel adenosinergic mechanism of regulation of TGFβ actions in the tumor microenvironment evident by the existence of cell-specific molecular loops: the TGFβ/CD73 axis on myeloid cells and the adenosine/TGFβ axis on fibroblasts. Decreased expression of CD73^+^ on myeloid cells in the tumor tissue of MMTV-PyMT mice correlated with increased collagen deposition and resulted in decreased tumor growth and metastasis. We found that adenosine was able to decrease TGFβ-induced phosphorylation of Smad proteins in fibroblasts *via* A2a/A2b receptors [37]. This work is in line with several studies that have previously demonstrated that TGFβ signaling can be regulated by cAMP-related pathways [38–41]. Based on our findings, we have suggested that adenosine, generated in the tumor microenvironment by myeloid cells, can shape TGFβ-depended effects of fibroblasts that contribute to the tumor growth and metastasis [37].

To elucidate how adenosine influences TGFβ-dependent fibroblasts functions during cancer progression, we have conducted experiments with mouse mammary fibroblasts and human fibroblast cell lines and have measured their proliferation, as well as secretory, migratory, and contractile fibroblast functions. Moreover, in co-culture experiments, we have confirmed that myeloid cells can affect functions of mammary fibroblasts through adenosine signaling.

## Materials and methods

### Genetically modified mice

Animal studies were approved by Vanderbilt Institutional Animal Care and Use Committee at Vanderbilt University Medical Center (IACUC M1600128-01). A2a-KO and A2b-KO animals were a gift from Dr. Igor Feoktistov (Vanderbilt University). Originally A2a-KO mice were obtained from Dr. Jiang-Fan Chen (Boston University) [42] and A2b-KO mice were obtained from Deltagen (San Mateo, CA) [43]. WT and CD73-KO mice were purchased from Jackson Laboratories, stock#000664 and #018986 respectively. CD39KO mice were provided by Dr. Simon Robson (Beth Israel Deaconess Medical Center and Harvard Medical School), where they were previously generated [44, 45].

### Cell lines and reagents

Immortalized mouse mammary fibroblasts were generated in Dr. Harold Moses laboratory (Vanderbilt University), have been used in previous publications [46–48], and gifted to us. Primary mouse mammary fibroblasts were isolated from mouse mammary gland by FACS sorting as described below. Fibroblasts were grown in T-75 flasks (Fisher Scientific, USA) in DMEM medium (Gibco, USA) supplemented with 10% FBS, Antibiotic-Antimycotic (Sigma-Aldrich). For protein assessment, fibroblasts were grown in 10cm cell culture dishes (Corning, USA). When cells achieved 90–95% confluency, they were treated with TGFβ, 1ng/ml (R&D System, USA) and 0.01, 0.1, 1, 10, 100 uM NECA (5′-N-Ethylcarboxamido adenosine, Sigma-Aldrich, USA). Following inhibitors were used: for inhibition of A2a adenosine receptors, 1 uM SCH58261(Tocris, USA); for A2b receptors– 1 uM PSB603 (Tocris, USA); for PLC inhibition– 1 uM U73122 (Tocris, USA); for PKA inhibition—1 uM H89 (Tocris, USA); and for activation of adenylate cyclase– 10 uM forskolin (Sigma-Aldrich, USA). Human fibroblast cell lines IMR-90 (CCL-186), WS-1 (CRL-1502), WPMY-1 (CRL-2584), hTERT SMC PM151T (“hTERT” CRL-3291) and BJ (CRL-2522) were purchased from ATCC and cultured according to the manufacturer’s protocol.

### Primary cell isolation

For isolation of mouse mammary fibroblasts, mouse mammary glands were resected and transferred into DMEM media (Gibco, USA) with 1 mg/ml Collagenase type I (Sigma-Aldrich, Worthington, MN, USA) and 1 mg/ml Dispase II (Roche Diagnostics, USA) for 2hr at 37°C. After incubation, single cell suspension was made and stained with antibody cocktail of anti- CD140a (PDGFRα)-PE, anti-CD45-FITC, anti-CD326 (Ep-CAM)-APC (Biolegend, USA). Fibroblasts were FACS sorted as CD140a^+^CD326^-^CD45^-^ cells using FACSAria sorter (BD).

For CD11b^+^ cell isolation, LLC tumors (3 weeks after s.c. injection) were resected and transferred into DMEM media (Gibco, USA) with 150 U/ml Collagenase type I (Sigma-Aldrich, Worthington, USA) and 100 U/ml Hyaluronidase (Sigma-Aldrich, Worthington, USA) for 1hr in 37°C. CD11b^+^ cells were isolated from single cell suspension using CD11b magnetic MicroBeads (Miltenyi Biotec, Germany). Purity of population was confirmed by flow cytometry.

### Western blotting

Protein lysates were generated from cells, scraped into RIPA buffer (Sigma-Aldrich, USA). Protein concentration was measured using BCA protein assay kit (Thermo Scientific, USA). Gel-electrophoresis was conducted on 4–12% NuPAGE gels (Invitrogen, USA) at 100V for 2hr. Proteins were transferred on 0.2 μm pore nitrocellulose membranes using iBlot gel transfer stacks (Invitrogen, USA), utilizing IBlot gel transfer device (Invitrogen, USA) at 20 V for 7min. Membranes were blocked with 2.5% milk (Bio-Rad, USA), diluted in TBS (Corning, USA) with 0.05% Tween 20 (Sigma-Aldrich, USA) for 1hr and incubated with following primary antibodies: anti-pSmad3 (Abcam, USA), anti-collagen I (Abcam, USA) and GAPDH (GeneTex, USA), diluted 1:7000 at 4°C overnight. Secondary HRP-conjugated anti-rabbit and anti-mouse antibodies (Promega, USA) were used in 1:30000 dilution for 2 hours at room temperature. Protein bands were detected with ECL reagent (Thermo Scientific, USA) on ChemiDoc imaging system (Bio-Rad, USA). Images were analyzed using ImageJ software (NIH).

### Analysis of cytokine secretion

Cell culture supernatants were assayed for CCL5, CXCL1, CXCL12, and VEGF concentrations by enzyme-linked immunosorbent assay kits (R&D Systems, USA) following manufacturer’s protocol.

### Proliferation assay

For proliferation assay, 10 000 fibroblasts/well of 96 well-plate were cultured for 24 hr in presence of TGFβ, NECA, or TGFβ+NECA. Fibroblast proliferation was assessed after the addition of 1 μCi (0.037 MBq)/well of [^3^H]-thymidine (GE Healthcare, USA) for 18 hours. All measurements were performed in triplicate and presented as mean CPM ± SE.

### Real-time PCR

Total RNA was isolated from cultured cells using RNeasy Mini kit (Qiagen, Germany) according to manufacturer’s specifications. cDNA was generated and subjected to quantitative RT-PCR using SYBR Green PCR Master Mix (Applied Biosystems, USA) using ABI PRISM 7900HT Sequence Detection System (Applied Biosystems, Foster City, USA) Primers used in RT-PCR were: *CCL5* forward: 5′-GCTGCTTTGCCTACCTCTCC-3′, reverse: 5′-TCGAGTGACAAACACGACTGC; *CXCL1* forward: 5′-CTGGGATTCACCTCAAGAACATC-3′, reverse: 5′-CAGGGTCAAGGCAAGCCTC-3′; *CXCL12* forward: 5′-TGCATCAGTGACGGTAAACCA-3′, reverse: 5′-TTCTTCAGCCGTGCAACAATC-3′; *VEGF* forward: 5′-CTGCCGTCCGATTGAGACC -3′, reverse: 5′-CCCCTCCTTGTACCACTGTC-3′; *αSMA* forward: 5′-GTCCCAGACATCAGGGAGTAA-3′, reverse: 5′- TCGGATACTTCAGCGTCAGGA-3′; *MMP9* forward: 5′–CTGGACAGCCAGACACTAAAG– 3′, reverse 5′-CTCGCGGCAAGTCTTCAGAG–3′; *β-actin* forward: 5′–GGCTGTATTCCCCTCCATCG– 3′, reverse: 5′–CCAGTTGGTAACAATGCCATGT– 3′. Relative mRNA expression in each sample was normalized to β-actin and presented using the comparative Ct method (2^ΔCt^).

### Gel contraction assay

The collagen gel was prepared from rat-tail collagen type I (Corning, USA), USA) at 3 mg/ml, 10 × Dulbecco’s Modified Eagle Medium (DMEM, Gibco, USA), 10 x NaHCO3, and sterile ddH_2_O. Immortalized mouse mammary fibroblasts (400,000 cells per assay); primary fibroblasts, isolated from mouse mammary gland (200,000 cells per assay); human fibroblasts cell lines (200,000 cells per assay); and CD11b^+^ cells, isolated from subcutaneous implanted LLC tumors (600,000 cells per assay), were embedded into collagen gels. Cell/gel mixture was placed in 24 well plates for 48 hr. Gels were treated with TGFβ (1 ng/ml), NECA (0.01, 0.1, 1, 10, 100 uM), AMP and ATP (both Sigma-Aldrich, USA) (100 uM) for 16–18 hr. After incubation, collagen gel plaques were detached from the bottom of the well for contraction evaluation. The change in collagen gel size (contraction index) was quantified with image analysis software (NIH ImageJ).

### Migration assay

Immortalized mouse mammary fibroblasts; primary mouse fibroblasts, isolated from mouse mammary glands were serum-starved (2% FBS, 12hr) and incubated with NECA and TGFβ for 16-18hr. After incubation, 30.000 cells were transferred onto cell culture inserts (polycarbonate membrane, 8um pore size, Corning, USA) and left for 3hr (immortalized mouse mammary fibroblasts) or 12 hr (primary mouse and human fibroblasts cell lines) to assess migration. The membranes from the inserts were stained with DAPI, imaged and number of cells was counted.

### Statistical analysis

Results were presented as Mean ± SEM. Multiple comparisons between groups were performed using one-way ANOVA followed by Dunnett’s procedure for multiplicity adjustment. Two-group comparison was performed using two-sample *t* tests or Wilcoxon Rank-Sum test as appropriate. The gene expression was normalized across all arrays. Gene symbols were assigned using the manufacturer-provided annotation, but the analysis was performed at probe level. Statistical analyses were performed using GraphPad Prism 8.0 Software (GraphPad Prism, USA) or R version 3.6.0

## Results

### Adenosine counteracts TGFβ-mediated effects on protein expression in mouse mammary fibroblasts

In our recently published work, in MMTV-PyMT mouse tumor model, we have determined that impact of TGFβ on CD73 expression on myeloid cells associates with the ability of adenosine to regulate TGFβ signaling on mammary fibroblasts [22]. We reported that this paracrine two-cell type (myeloid cell—fibroblasts) negative feedback loop mechanism may contribute to a novel adenosinergic mechanism of TGFβ regulation in the tumor microenvironment and represents a potential therapeutic target. While we have observed changes in ECM deposition that correlated with decreased tumor growth and metastasis [37] in MMTV-PyMT mouse tumor model, the role of adenosine in the regulation of broad TGFβ-dependent fibroblast functions—migration, contraction, and protein secretion need to be clarified.

To determine how adenosine can modulate gene expression profile of fibroblasts, we have assessed CCL5 (RANTES), CXCL1 (KC), CXCL12 (SDF1a), MMP9 (matrix metalloproteinase-9), aSMA (alpha-smooth muscle actin), and VEGF (vascular endothelial growth factor). These proteins are known regulators of tumorigenesis and we have successful studied them in the past [22, 49–57].

**Fig 1:**
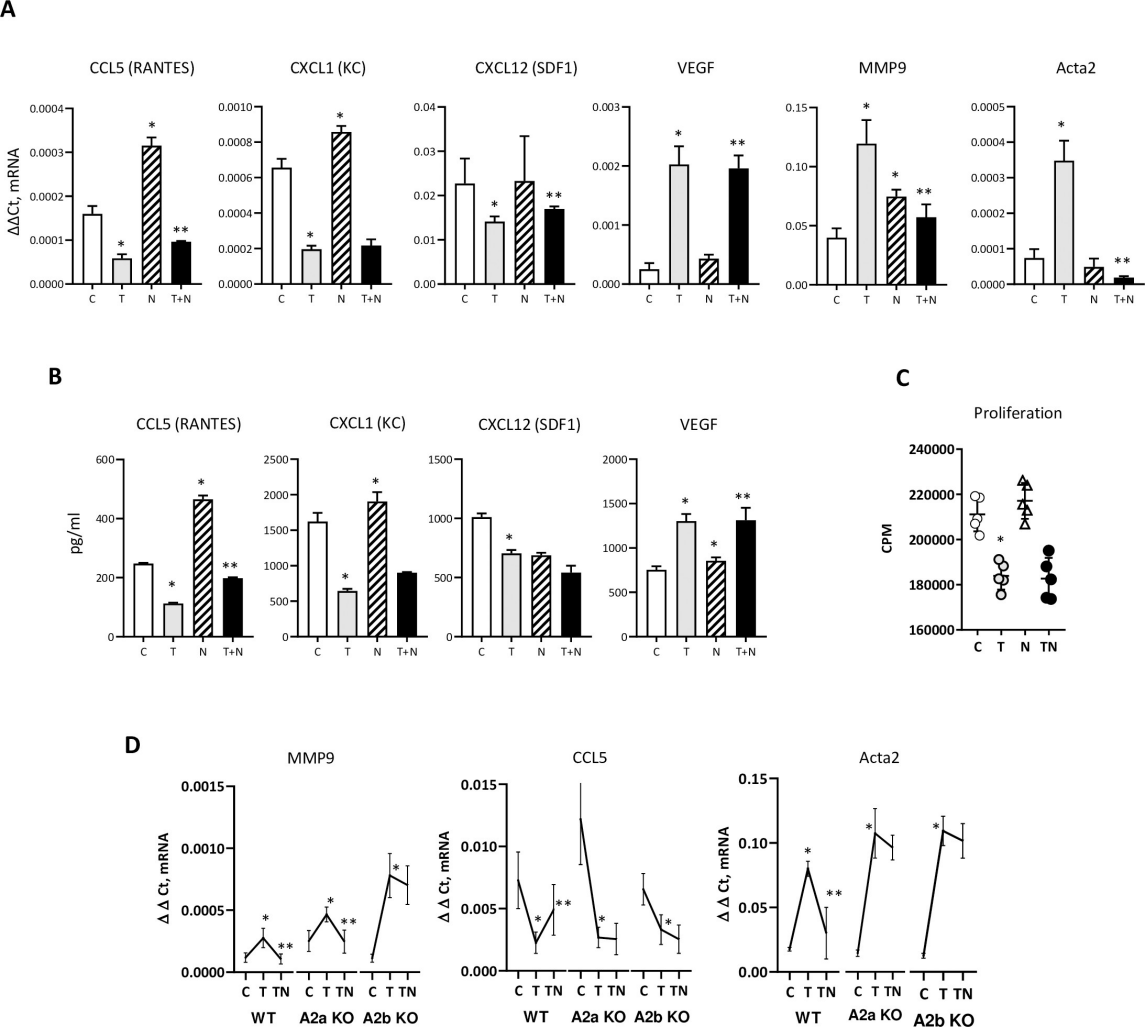
Effect of adenosine on TGFβ dependent proteins. **A**) qPCR analysis of immortalized mouse mammary fibroblasts. Graphs show Δct expression of *CCL5(RANTES)*, *CXCL1(KC)*, *VEGF*, *MMP9*, and *αSMA*. *—p<0.05 vs. control (C) group, **—p<0.05 vs. TGFβ (T) group. Mouse mammary fibroblasts were cultured with TGFβ (1 ng/ml) and/or NECA (10 uM) for 18 hr. with following RNA isolation for qPCR analysis. **B**) Secreted protein measurement by ELISA. Cells (5x10^5^) in 6 wells plate/3 ml of medium were cultured overnight. *—same as in A. **C**) Proliferation assay (thymidine-[H3]) of mouse mammy fibroblasts. Graph shows CPM counts. Mouse mammy fibroblasts were cultured 24 hr. with TGFβ (1 ng/ml) and/or NECA (10 uM) and presence of 1 uCi. C- control, T–TGFβ, N–NECA, T+N–TGFβ + NECA. **D**) qPCR analysis of primary mouse mammary fibroblasts isolated from mouse mammary gland of WT and A2a-KO, and A2b-KO animals. Graphs show Δct expression of *CCL5*(RANTES), *MMP9*, and *αSMA*. Primary mouse mammary fibroblast isolated by FACS sorting as CD104a^+^ (CD45^-^CD326^-^) cells. Cells were used in passage 1 and incubated with TGFβ and/or NECA.

We have incubated mouse mammary fibroblasts with TGFβ, NECA and TGFβ+NECA and measured gene expression (**Fig 1**). NECA was used in 10 uM concentration as previously [58], resembling the level of adenosine in solid tumors [59]; this concentration activates both A2a and A2b adenosine receptors. We found that NECA counteracts TGFβ inhibitory effect on *CCL5*, *CXCL12*, but lessens upregulated *MMP9* (**Fig 1A**). Interestingly, we observed that NECA, exhibits no effect on αSMA *(ACTA2)* by itself, was able to completely abolish TGFβ-induced αSMA upregulation (**Fig 1A**). We followed our studies with ELISA assays to confirm RT-PCR data and found same changes for CCL5 and CXCL1 proteins (**Fig 1B**). While we have observed similar tendency for CXCL12 and VEGF, these changes have not reach significance in NECA + TGFβ group vs. TGFβ only. Next, in proliferation assays, we have not found that NECA is able to modulate TGFβ effect on fibroblast proliferation (**Fig 1C**). Finally, to dissect adenosine receptor subtype, responsible for mentioned above effects on TGFβ, we have utilized mammary fibroblasts, isolated from WT, A2a-KO, and A2b-KO mice. We observed that effects on *CCL5* and *ACTA2* were regulated by both A2a and A2b adenosine receptors, whereas effects on expression of *MMP9* was modulated through A2b adenosine receptors (**Fig 1D**).

### Adenosine changes TGFβ induced mammary fibroblasts migration and gel contraction

**Fig 2:**
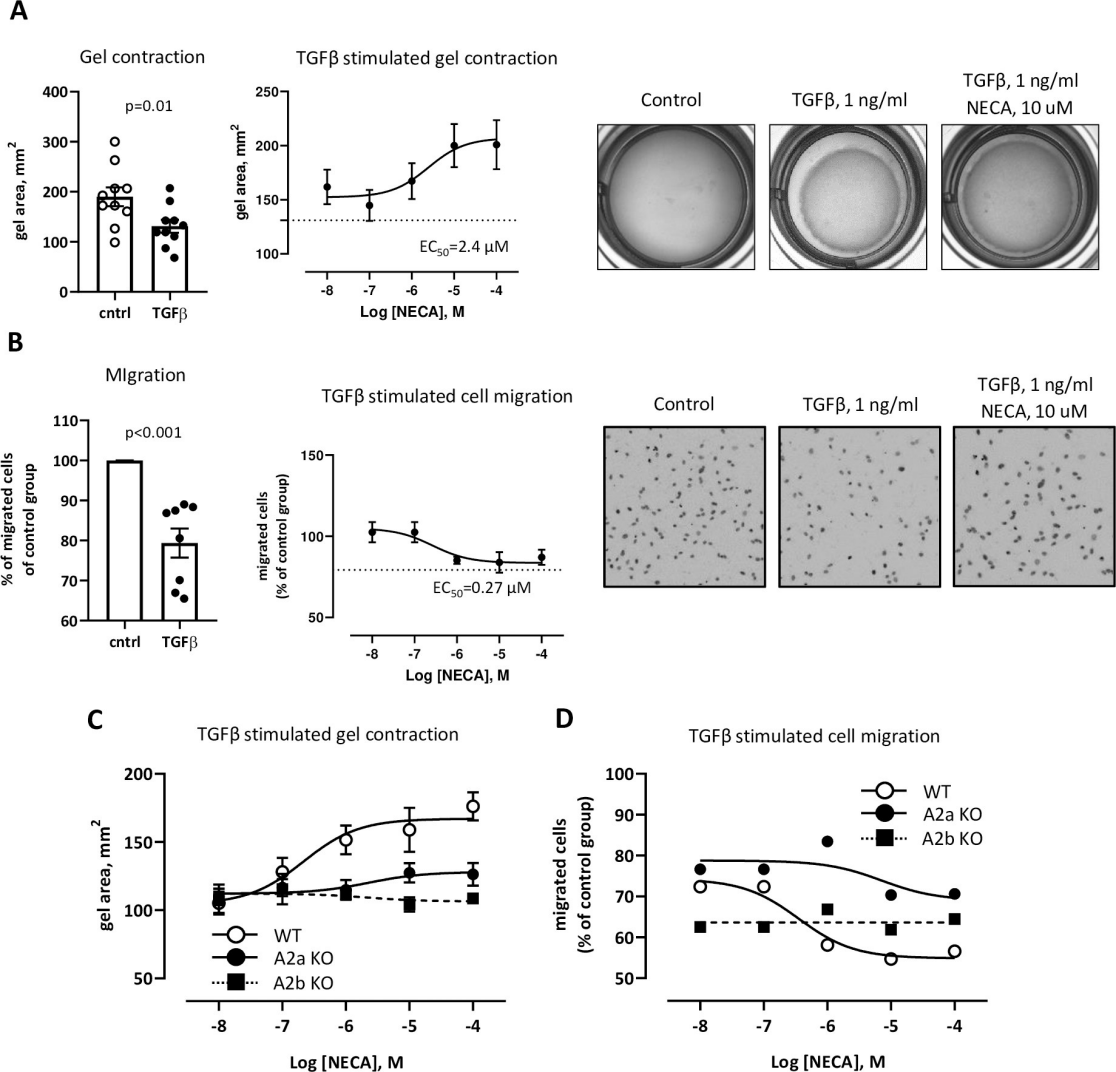
Effect of adenosine on fibroblast migration and gel contraction. **A**) Gel contraction assay of mouse mammary fibroblasts. Graphs show gel area, mm^2^. Left plot demonstrates control vs. TGFβ. *—p<0.05 vs. control. Right, logged plot, demonstrates NECA influence on TGFβ induced effect. Mouse mammary fibroblasts were embedded to collagen gel and cultured 24 hr. TGFβ (1 ng/ml) was added for 6 hr. with following measurement of gel diameter. For dose-dependent experiments NECA (0.01 uM– 100 uM) was added together with TGFβ. Dotted line is a TGFβ only. **B**) Migration assay of mouse mammary fibroblasts. Graphs show % of migrated cells of control group which is used as 100%. Left plot demonstrates control vs. TGFβ. *—p<0.05 vs. control. Right logged plot demonstrates NECA influence on TGFβ induced effect. Mouse mammary fibroblasts were placed on the top of 24 wells insert and cultured for 3 hr. in presence of TGFβ (1 ng/ml) and NECA (0.01 uM– 100 uM). Dotted line is a TGFβ only. **C**, **D**) Logged plots of gel contraction and a migration assays of primary mouse mammary fibroblasts isolated from mouse mammary gland WT and A2a-KO, and A2b-KO animals. Graphs show gel area, mm^2^ for gel contraction or % of migrated cell over the control group. TGFβ and NECA concentrations are the same as in A and B.

We found that NECA affects TGFβ induced *αSMA* expression (**Fig 1A**) indicating that motility and contractility of fibroblasts can be regulated through adenosine/TGFβ axis. To estimate the contribution of TGFβ and NECA to these processes we have performed gel contraction and migration assays using mouse mammary fibroblasts. We found that NECA decreases TGFβ- induced gel contraction with EC50 2.4 uM (**Fig 2A**). Migration assay revealed that TGFβ downregulates migratory ability, but NECA with EC50 0.27 uM counteracts this effect (**Fig 2B**). Considering 10-fold difference in EC50 for these two assays we have hypothesized that different types of adenosine receptors are involved in regulation of TGFβ-mediated migration and contraction of fibroblasts. To check this hypothesis, we have utilized primary mammary fibroblasts, isolated from WT, A2a-KO, and A2b-KO mice. We demonstrate that TGFβ-induced gel contraction was modulated by NECA *via* A2b adenosine receptors, whereas migration was regulated through both A2a and A2b adenosine receptors (**Fig 2C and 2D**). Overall, TGFβ/adenosine axis regulates not only secretory functions of mammary fibroblasts but is involved in modulation of their migratory and contractility abilities as well. In addition, this mechanism demonstrates dependency on concentration of adenosine and can be mediated through different subtypes of adenosine receptors.

### Adenosine regulates TGFβ actions on mammary fibroblasts through adenylyl cyclase—protein kinase A dependent mechanism

**Fig 3:**
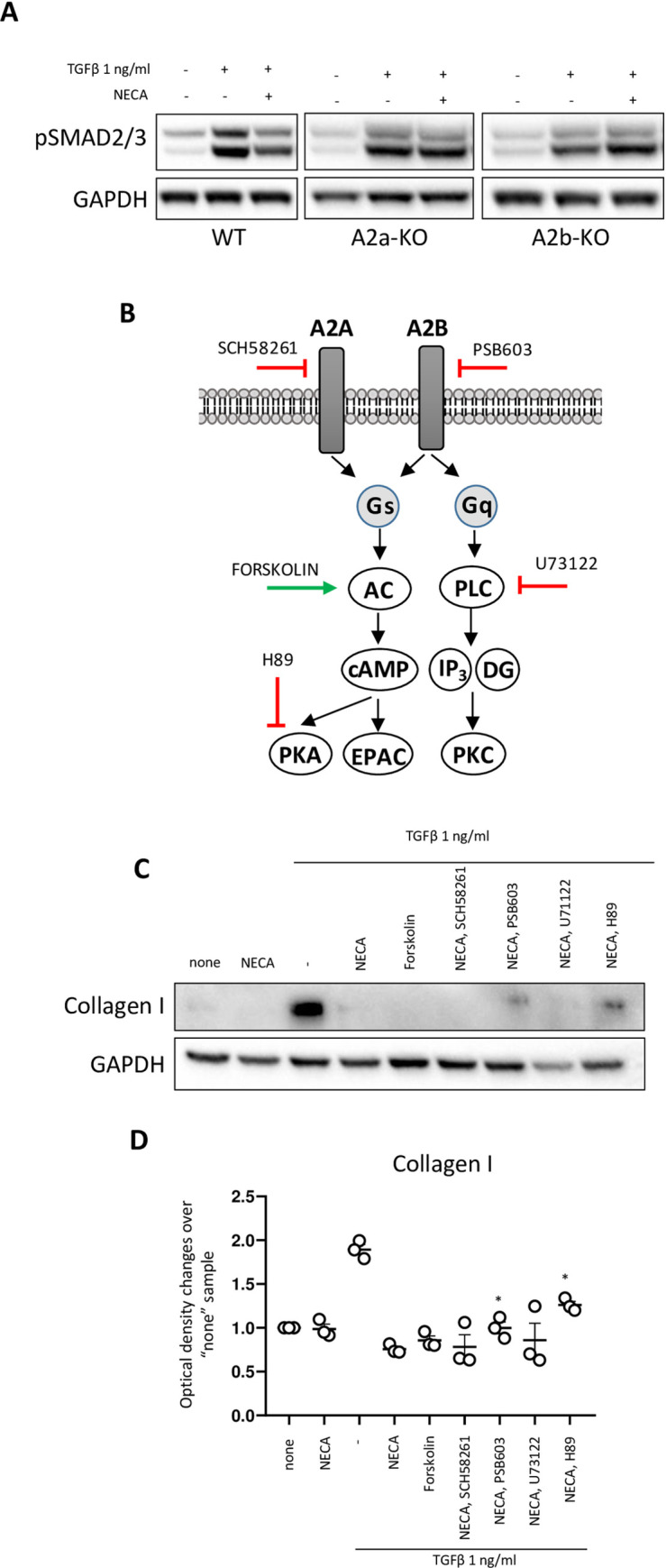
Molecular mechanism of adenosine in regulation TGFβ signaling. **A**) Representative Western blotting for pSMAD3 in mouse mammary fibroblast isolated from WT, A2a-KO, and A2b-KO mice. **B**) Scheme of adenosine signaling through A2a and A2b receptors. Targets for activation/inhibition of key components of signaling pathway are indicated. **C**) Representative Western blotting for Collagen (intracellular) and **D**) Quantitative analyzes of Western blotting using Image J software from 3 different experiments. *—p<0.05 compare to “TGFβ +NECA group”.

In our previous work, we have demonstrated that adenosine directly impacts TGFβ signaling evident by reduced pSmad 2/3, a major complex of TGFβ canonical signaling pathway [37]. To dissect further the receptor subtype, mediated this effect, we show that wild type mouse mammary fibroblasts, incubated with TGFβ in presence of NECA do not respond by Smad phosphorylation, whereas A2a-KO and A2b-KO fibroblasts retain this response (**Fig 3A**). To continue to evaluate the molecular interplay between adenosine and TGFβ signaling pathways we have investigated influence of NECA on TGFβ-induced synthesis of collagen 1 (Col I) utilizing pharmacological approach. We have used WT primary mammary fibroblasts and antagonists of A2a (SCH58261) and A2b (PSB603) receptors. Western blot assay demonstrated that TGFβ induced Col I synthesis affected through A2b receptor (effect PSB603). To look at downstream of Gs and Gq signaling that mediates through adenylyl cyclase (AC) or phospholipase C (PLC) cascade (**Fig 3B**), we have used activator of AC (Forskolin) and inhibitors of PLC (U73122) and protein kinase A (PKA) (H89). Forskolin recapitulated effect of NECA, blocking TGFβ ability to stimulate Col I synthesis. We found that PSB603 and H89 were able to recover some of TGFβ-induced Col I synthesis in presence of NECA, whereas SCH58261 did not show this effect (**Fig 3C and 3D**). These pharmacological studies demonstrate that the adenosine influences TGFβ actions through activation of adenylyl cyclase and activation of PKA.

### Adenosine-producing myeloid cells affect mechanical properties of mammary fibroblasts

**Fig 4:**
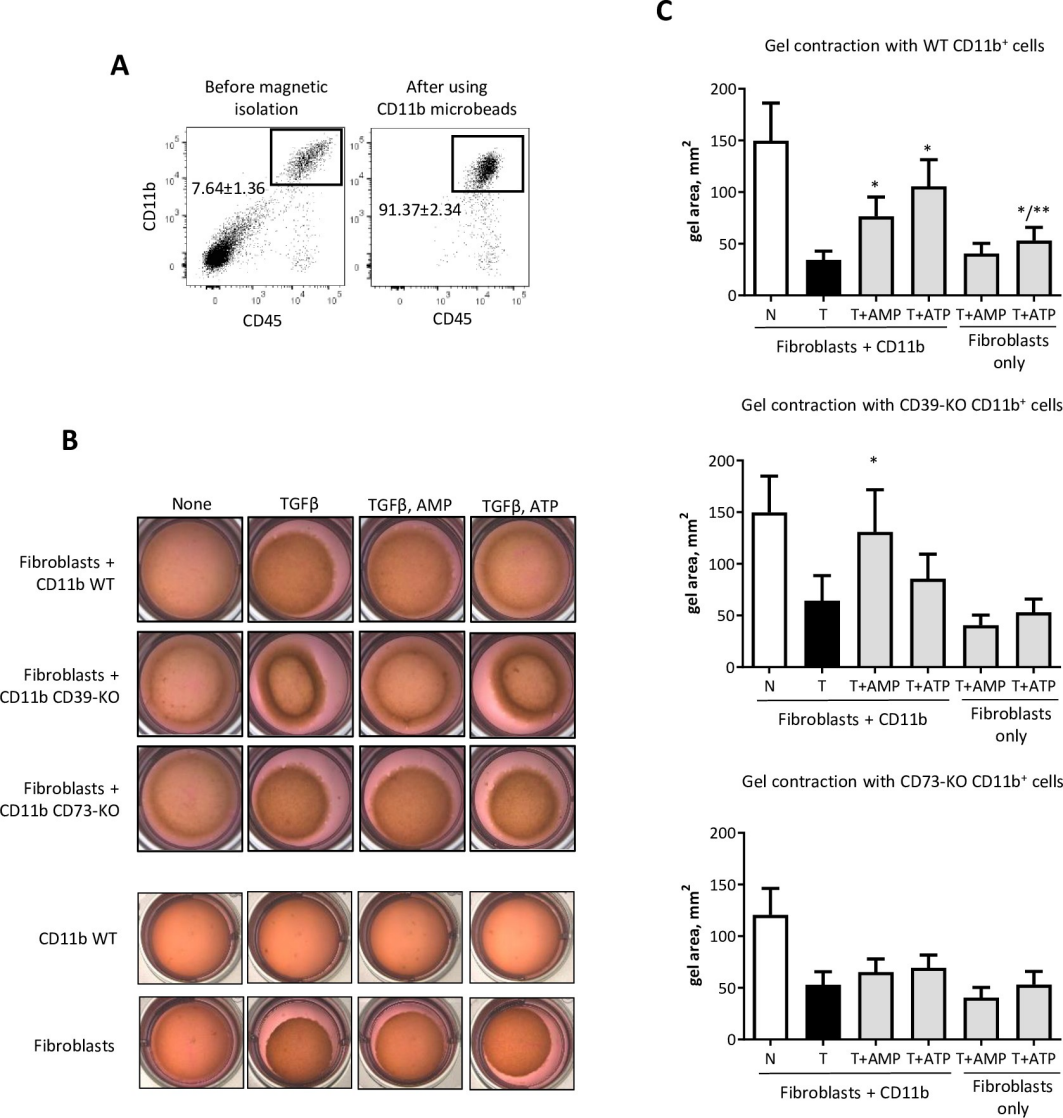
Role of myeloid cells on gel contraction. **A**) FACS plots (DAPI^-^) before and after isolation of CD11b^+^ cells from s.c. LLC tumor tissue using CD11b magnetic microbeads. Gate (CD45^+^CD11b^+^) indicates average purity of isolated cells based on 5 separate experiments. **B**) Representative gel pictures after performing gel contraction assay. **C**) Gel contraction assay of immortalized mouse mammary fibroblasts co-incubated with CD11b^+^ cells. Graphs show gel area, mm^2^. *—p<0.05 vs. TGFβ group. Primary mouse mammary fibroblast isolated from mouse mammary gland WT animals by FACS sorting as CD104a^+^ (CD45^-^CD326^-^) cells. Cells were used in passage 1. CD11b cells were isolated from LLC implanted tumors by magnetic microbeads. Fibroblasts and CD11b cells were embedded to collagen gel and cultured 24 hr. TGFβ (1 ng/ml) and AMP/ATP (100 uM) was added for 6 hr. with following measurement of gel diameter.

In our previous work, we have postulated that CD73^+^ myeloid cells can regulate TGFβ dependent functions of fibroblasts by supplying the tumor microenvironment with adenosine [37]. Here, we have measured mouse mammary fibroblasts function in a gel contraction assay in respond to co-incubation with CD11b^+^ cells, isolated from LLC tumors that were s.c. implanted to WT, CD39-KO and CD73-KO mice (**Fig 4A**) in presence of ATP (substrate for CD39) and AMP (substrate for CD73). Our preliminary data showed that effective concentration of ATP and AMP for gel contraction assay is 100 uM. Myeloid cells (CD11b^+^) alone do not contract gel with or without ATP/AMP incubation (**Fig 4B**). Fibroblasts alone respond to TGFβ by gel contraction with no effect by AMP, but slightly inhibitory effect by ATP (**Fig 4B**). We observed that co-incubation fibroblasts with CD11b^+^ cells with AMP and ATP reduces gel contraction in comparison to TGFβ group. In a group of mammary fibroblasts, co-incubated with CD39-KO CD11b^+^, we have observed reduction of contractility function only in presence of AMP, whereas in group with CD73-KO CD11b^+^ cells no changes were seen in presence of AMP or ATP (**Fig 4C**). Taken together, these data demonstrate that presence of CD39^+^CD73^+^ myeloid cells in the tumor microenvironment can metabolize extracellular ATP, ADP and AMP into adenosine, which, in turn, can shape TGFβ- induced functions of fibroblasts.

### Adenosine diminish TGFβ effects on human fibroblasts

**Fig 5:**
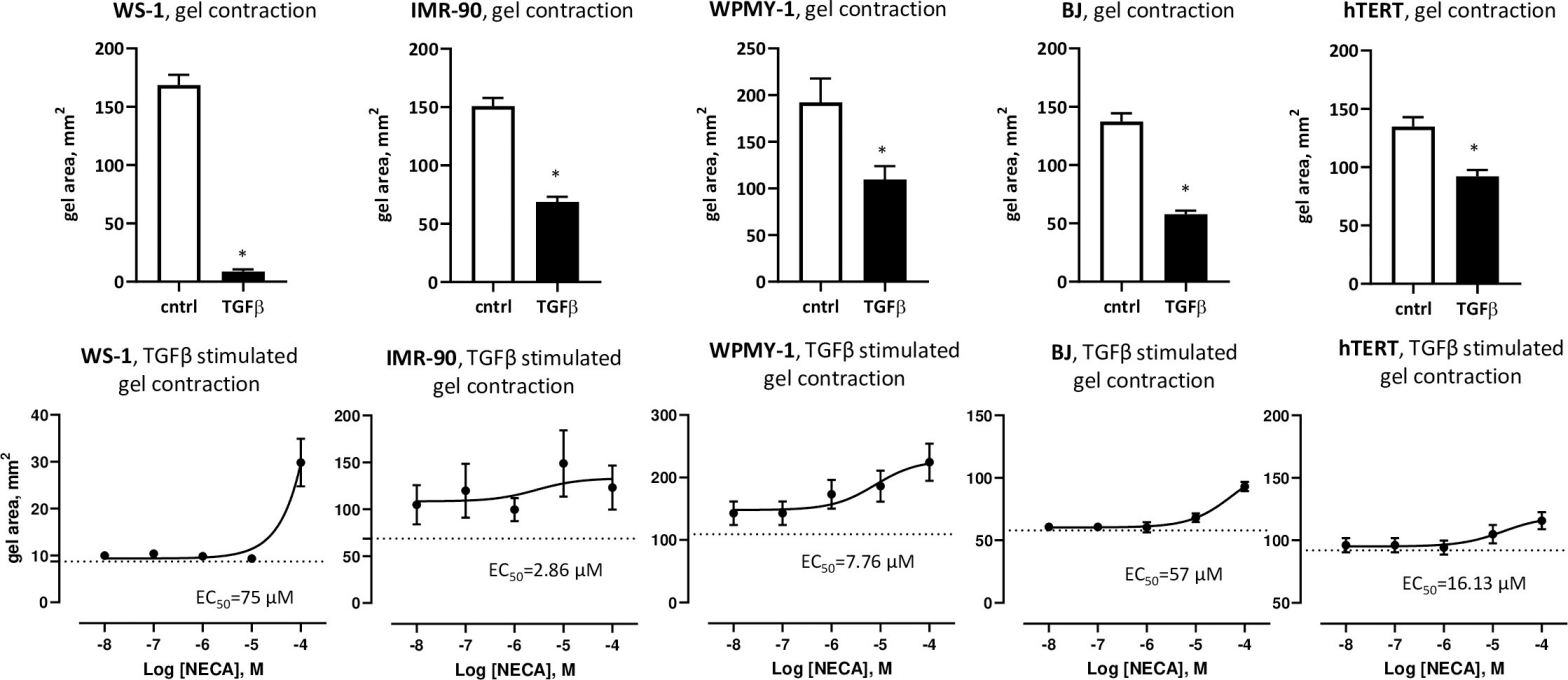
Adenosine/TGFβ interaction in human fibroblasts. Gel contraction assay of human fibroblast cell lines. Graphs show gel area, mm^2^. Top raw demonstrates control (cntrl) vs. TGFβ (1 ng/ml). *—p<0.05 vs. control. Cell lines were embedded to collagen gel and cultured 24 hr. TGFβ (1 ng/ml) was added for 6 hr. with following measurement of gel diameter. Bottom raw, logged plot, demonstrates NECA influence on TGFβ induced effect. Cell lines were embedded to collagen gel and cultured 24 hr. TGFβ (1 ng/ml) and NECA (0.01 uM– 100 uM) were added in same time for 6 hr. with following measurement of gel diameter. Dotted line is the TGFβ only.

To determine if we can recapitulate the findings of adenosine impacting TGFβ actions on mouse mammary fibroblasts into human fibroblasts, we have used all available to us commercial human fibroblast cell lines: IMR-90 (lung), WS-1 (skin), BJ (skin, foreskin), hTERT (prostate), and WPMY-1 (prostate/stroma). In these comparative studies, we have performed gel contraction assay. We found that all mentioned above cell lines were responding to TGFβ by gel contraction (**Fig 5**, top panel), which was reduced in presence of adenosine (NECA) (**Fig 5**, bottom panel) by 1.8 fold (IMR-90), 3.5 fold (WS-1), 1.6-fold (BJ), 1.25-fold (hTERT) and 2.1-fold (WPMY-1). This effect is comparable to 1.5-fold decrease, observed in mouse mammary fibroblasts (**Fig 2**). We found selective sensitivity to adenosine among different cell lines with IMR-90 cell line being a most sensitive (EC50 2.86 uM) and WS-1 cells line—a least sensitive (EC50 75 uM). Interestingly, we found IMR-90 was responding to the whole dose range of NECA with similar, albeit, variable response (at the lowest NECA 10^−8^ M: 1.5 fold change from baseline (TGFβ only), at the highest NECA 10^−4^ M: 1.8 fold change). Altogether these experimental results, combined with data presented in Fig 2, are suggestive that modulatory effect of adenosine to TGFβ on human fibroblasts is mediated through low affinity A2b adenosine receptor.

## Discussion

In the current work we have provided analysis of the interaction between adenosine and TGFβ signaling and its impact on fibroblast function. Our group has reported that adenosine modulates TGFβ-regulated protein expression. In addition, we demonstrated that adenosine regulates TGFβ-induced cytoskeletal rearrangements and affects the migratory and contractility functions of fibroblasts. Additional experiments have shown that the observed changes are mediated through A2a/A2b subtypes of adenosine receptors. We have confirmed our hypothesis that myeloid cells in the tumor microenvironment by generation of adenosine from extracellular ATP/AMP, can regulate TGFβ-induced functions on fibroblasts through adenosine receptors. A more detailed investigation of the crosslink between adenosine and TGFβ signaling pathways revealed a critical role of AC-PKA. Furthermore, we have reported that these modulatory effects of adenosine/TGFβ axis are evident in both murine and human fibroblasts.

In cancer, tissue fibroblasts play diverse roles and participate in many processes related to the tumor cell invasion and metastasis. Fibroblasts synthesize a wide variety of cytokines, chemokines, enzymes, growth factors and matrix proteins that affect tumor microenvironment composition and activity [60]. We found that NECA increases expression of *CCL5* that was downregulated by TGFβ. An *et al*., reported that *CCL5* in the tumor microenvironment is associated with migration and invasion of tumor cells and macrophage phenotype alteration towards pro-tumorigenic [61]. Secretion of CCL5 by fibroblasts in the tumor tissue is associated with changes of tumor cell behavior to more aggressive, the recruitment of immunosuppressive cells, and the activation of angiogenesis [62].

Fibroblasts play a key role in the development of tumor ECM by synthesis, folding, and secretion of a wide variety of ECM proteins such as collagen, fibronectin, and laminin. Many reports indicate that CAFs participate in ECM remodeling acting predominantly as pro-tumorigenic agents [60]. We have discovered a significant reduction of TGFβ-induced expression of *MMP9* by NECA. MMP9 participates in ECM remodeling, alteration of cell-cell and cell-ECM interactions, and cleavage of membrane surface proteins and is associated with poor prognosis in cancer patients [55]. One of the most important pro-tumorigenic characteristics of ECM in the tumor microenvironment is providing mechanical signals for cancer cells and formation of invasion tracks [63]. Current research work has elucidated that adenosine reduces the expression of *αSMA* that was upregulated by TGFβ. In addition, a gel contraction assay revealed that NECA reduces TGFβ-activated contraction. During tumor progression, fibroblasts undergo phenotype alterations and are characterized by upregulated *αSMA* expression. Expression of *αSMA* correlates with contractility of CAFs, which is crucial for mechanical ECM remodeling and is associated with tumor cell invasion and metastasis [3, 64]. From another hand, fibroblasts can directly contribute to metastasis by direct involvement into group migration and invasion [65–69]. Here, we report that NECA can overwrite TGFβ effect on fibroblast migration upregulating this function. This TGFβ aberrant respond can be associated with the aggressive behavior of tumors with reduced TGFβ signaling on fibroblasts [70].

Our data suggest that adenosine regulates TGFβ-mediated functions of fibroblasts mostly through A2a or/and A2b adenosine receptors. Purinergic signaling is a very complex system and includes diverse receptors that are characterized by differing expression on cell types, affinity to their ligands, and downstream signaling machinery [71, 72]. The P1 group includes four G-protein coupled adenosine receptors: A1, A2a, A2b, and A3, that are characterized by different affinities to adenosine. A1, A2a, A3 receptors transduce high affinity (EC_50_ in range of 0.1–0.7 uM) signals, while A2b receptors were suggested as low affinity receptors due they responsiveness to high concentrations of adenosine (EC_50_ in range 1–100 uM). Importantly, latter conditions are observed only under pathological settings, including in the tumor microenvironment [71]. Indeed, our results suggest that the contractility of fibroblasts can be modulated through A2b adenosine receptors, while the migratory function is regulated through A2a and A2b receptors. TGFβ stimulated MMP9 expression was modulated through A2b receptors, at the same time expression of CCL5 and αSMA was controlled through both A2a and A2b receptors. In our previous work we have shown that adenosine can reduce the amount of TGFβ-induced pSmad3 in mammary fibroblasts [37]. In this current work we have further analyzed TGFβ signaling and have demonstrated that adenosine can modulate the phosphorylation of Smad and the synthesis of collagen in A2b-AC-PKA dependent manner. Forskolin, a well-known activator of AC, mimics the effects of NECA and demonstrates a reduction of Col I synthesis induced by TGFβ.

Morphogens like TGFβ, Wnt and Sonic Hedgehog are characterized by a complex mechanism of signaling transduction. In addition, their signaling pathways demonstrate multiple crosslinks with each other and with signaling pathways of different active compounds. We have demonstrated that the interaction between TGFβ and adenosine signaling pathways is mediated in an A2a/A2b-AC-PKA dependent manner. Interestingly, Zhang *et al*., have reported that the activated Smad3/Smad4 complex can bind to the regulatory subunit of PKA holoenzyme, form a trimeric complex, and result in the activation of PKA without changing the cAMP concentration [73]. Decreased TGFβ-induced Smad3 phosphorylation in presence of NECA can be caused by interruptions in TGFβ receptor binding, Smad2/3 complex interactions or in direct dephosphorylation, ubiquitination, and subsequent proteasome degradation of pSmad3. TGFβ receptor activation can be regulated by a wide variety of intracellular proteins like BAMBI, FKBP12, Smurf, or Smad7. Smad6 and Smad7 (I-Smads) compete with R-Smads for receptor, Co-Smad binding, or participate in receptor degradation and their activation negatively regulates TGFβ signaling [74]. Smurf E3 ligases affect several levels of the TGFβ signaling pathway, promoting degradation of R-Smads, I-Smads and TGFβ receptors. Moreover, Smurf2 interacts with Smad7 to promote its binding with the activated TGFβ receptor complex, which leads to proteasome degradation of both activated TGFβRI and Smad7 [75, 76]. We can speculate that there could be an undescribed mechanism of AC-cAMP-PKA regulation of Smurf activity that can affect Smad signaling. R-Smads can be dephosphorylated with subsequent degradation [74]. Adenosine signaling can induce phosphatase activity increasing Smad2/3 dephosphorylation. TGFβ induces production of Col I, FN and CTGF by activation of Smad2, Smad3, ERK12, and p38 MAPK phosphorylation. This fact indicates that the A2a/A2b-AC-cAMP-PKA pathway affects the non-canonical shoulder of TGFβ signaling as well.

In this current work, we have demonstrated that myeloid cells, isolated from mouse tumor tissue and co-cultured with mammary fibroblasts, generate adenosine which modulates TGFβ-stimulated gel contraction. The ATP/ADP release into extracellular space can be caused by disrupting the cell integrity or by active secretion. Under normal conditions, accumulation of nucleotides and nucleosides in tissue is minimal and is essential for normal cell functioning [77]. However, during various pathological condition their concentrations in extracellular space can be extreme [77]. Extracellular ATP and ADP signaling is limited by degradation rate that is depends upon expression and activity of several membrane linked ectonucleotidases [71], including ectonucleoside triphosphate diphosphohydrolases (CD39, CD39L, CD39L3), ectonucleotide pyrophosphatase/phosphodiesterase CD203a (NPP1), alkaline phosphate (ALP). Resulting metabolite of ATP/ADP hydrolysis is AMP that has no own receptor and hydrolyzes into adenosine by ecto-5′-nucleotidase (NT5, CD73) [71, 72]. Our group have demonstrated several types of cells that express CD39 and CD73 in the tumor microenvironment: immune cells (myeloid cells, T-cells), fibroblasts, endothelial cells, and neoplastic cells. In addition, we have identified terminally differentiated myeloid cell (TDMC), that co-express CD39 and CD73 and are a primary source of adenosine in the tumor tissue in a model of breast cancer [22]. Number of research works demonstrated critical role of CD39 and CD73 in tumor development and patients’ survival [78]. In past, we have showed that downregulated expression of CD73 on myeloid cells correlated with reduced tumor growth and metastasis. Intriguing observation in this work was enhanced deposition of collagen in the tumor tissue revealed by histological analysis. We hypothesized that adenosine, produced by CD39^+^CD73^+^ cells can modulate TGFβ signaling on fibroblasts and change their ECM producing ability [37]. In the current work, we are providing more detailed information about the role of adenosine in controlling TGFβ-mediated fibroblast function. We found that adenosine regulates secretory, migratory and contractility functions of fibroblasts in an A2a and/or A2b dependent manner. The microenvironment of solid tumors is often associated with a high concentration of adenosine [79] corresponding to activation of low affinity A2b subtype of adenosine receptors. We have determined that MMP9 expression and contractility function of fibroblasts appear to be regulated solely by A2b receptors. In summary, the data outlines A2b adenosine receptors as mediators of adenosinergic regulation of TGFβ actions on fibroblasts.

To test the hypotheses that discovered mechanism is common for different types of fibroblasts and to demonstrate its transability to human, we have used cell lines generated from human lung (IMR-90), skin (WS-1, BJ), and prostate (WPMY-1, hTERT SMC PM151T) fibroblasts. TGFβ signaling in cancer-associated fibroblasts plays an important role for their activation, motility, contractile function, production of anti/pro-tumorigenic factors, and ECM synthesis and remodeling. On the other hand, there is an evidence that lack of TGFβ signaling in fibroblasts is correlated with poor cancer prognosis. Cheng et al. demonstrated that loss or lack of TGFβ signaling in mammary cancer associated fibroblasts increase tumor growth and metastasis [25, 47]. Oyanagi et al. showed that inhibition of TGFβ signaling in fibroblasts can enhance migratory and invasive activity of Panc-1 cells in the model of pancreas cancer [80]. Because origin of cancer tissue determines tumor development patterns, function of fibroblasts and TGFβ signaling in this type of cells can be regulated by different mechanisms. Modulation of TGFβ effects can be realized in different levels of signal transduction: regulation of accessibility of TGFβ ligand to its receptor, insufficient downstream signaling, and crosslink with other signaling pathways [74, 81–85]. Gel contraction assay has demonstrated ability of adenosine to decrease TGFβ induced contractility in different types of fibroblasts. Further investigation needed to elucidate the role of discovered phenomenon in progression of different types of cancer.

The pleotropic functions of TGFβ during cancer progression is determined by the interplay with other signaling pathways that are activated in the tumor microenvironment or under other pathological conditions. Our data suggests a novel mechanism of adenosine/TGFβ signaling pathways interaction that defines CAFs phenotype in the tumor microenvironment. Current research work proposes the A2b receptor on fibroblasts as a target for anticancer therapy and can be used as a basis for the development of novel therapeutic approaches.

## Supporting information

S1 Raw images (PDF)
