# Supplementary figures and images for: Adenosine/TGFβ axis in regulation of mammary fibroblast functions
Source: PLoS One. 2021 Jun 8;16(6):e0252424. doi: 10.1371/journal.pone.0252424 (PMC8186761; doi:10.1371/journal.pone.0252424)

### A, left (WT)

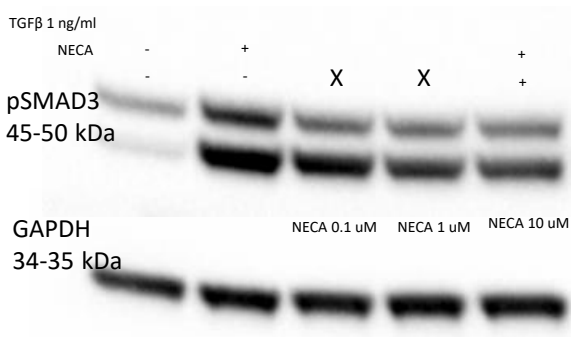

### A, center (A2a-KO)

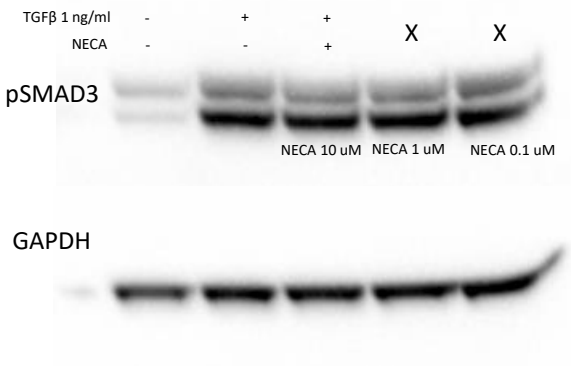

### A, right (A2b-KO)

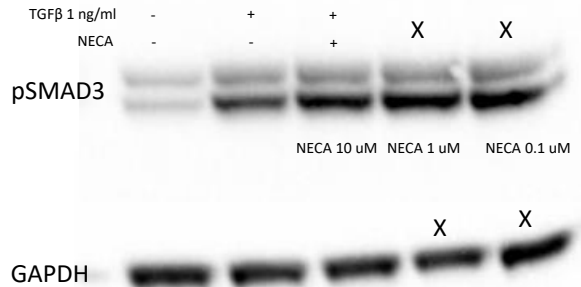

### C

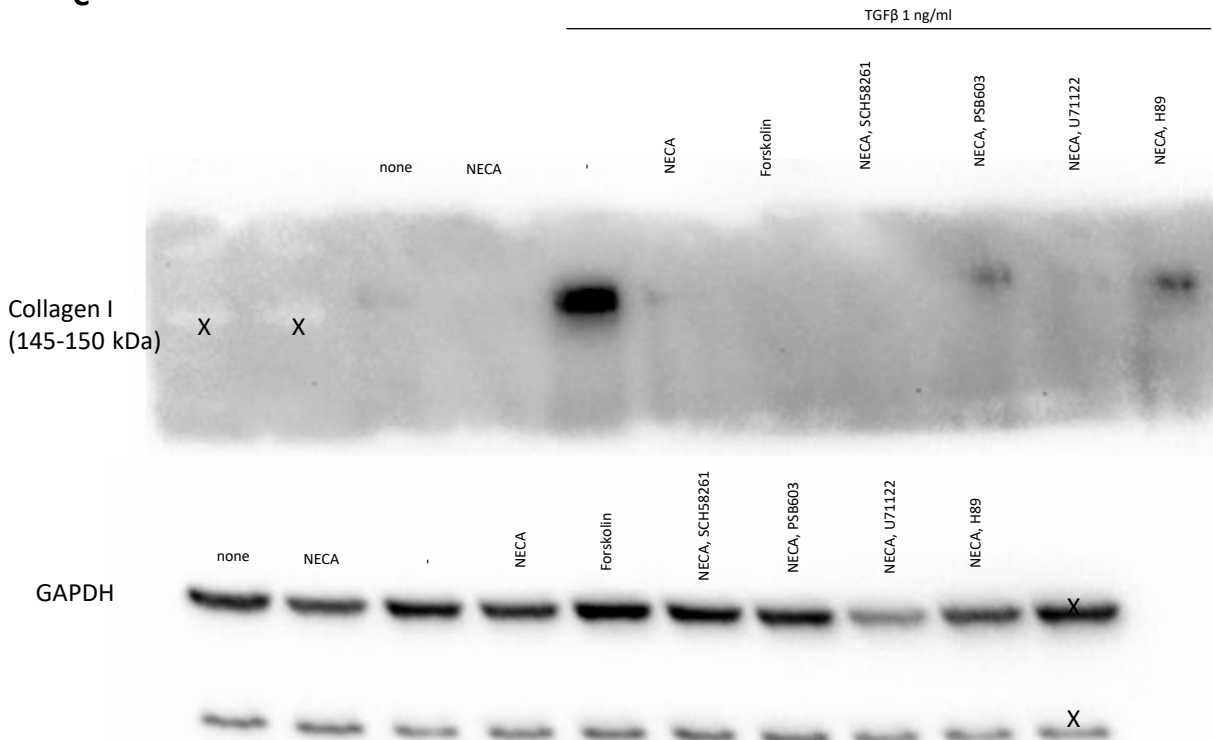

Supplement: S1 Raw images — (PDF) [file pone.0252424.s001.pdf]
